# The Effects of 32 Weeks of Multicomponent Training with Different Exercises Order in Elderly Women’s Functional Fitness and Body Composition

**DOI:** 10.3390/medicina58050628

**Published:** 2022-04-30

**Authors:** António M. Monteiro, Sandra Rodrigues, Sérgio Matos, José E. Teixeira, Tiago M. Barbosa, Pedro Forte

**Affiliations:** 1 Research Centre in Sports Sciences, Health and Human Development (CIDESD), 5001-801 Vila Real, Portugal; jose.eduardo@ipb.pt (J.E.T.); barbosa@ipb.pt (T.M.B.); 2 Department of Sport Sciences and Physical Education, Instituto Politécnico de Bragança (IPB), 5300-253 Bragança, Portugal; 3 FP-I3ID, FP-BHS, Escola Superior de Saúde Fernando Pessoa, Rua Delfim Maia, 334, 4200-253 Porto, Portugal; sandrar@ufp.edu.pt; 4 Polytechnic Institute of Viana do Castelo, School of Sport and Leisure, 4960-320 Melgaço, Portugal; sergioms@esdl.ipvc.pt; 5 Department of Sports, Higher Institute of Educational Sciences of the Douro, 4560-708 Penafiel, Portugal; 6 Research Center in Sports Performance, Recreation, Innovation and Technology (SPRINT), 4960-320 Melgaço, Portugal; 7 CI-ISCE, Higher Institute of Educational Sciences of the Douro, 4560-708 Penafiel, Portugal

**Keywords:** women, exercises, order, multicomponent, training

## Abstract

*Background and objectives:* Starting the multicomponent training sessions with aerobic-based exercises or resistance-based exercises may have different effects on functional fitness and body composition. Thus, the aim of this study was to assess the effects of the order of exercises in elderly women’s physical fitness and body composition by multicomponent training. *Materials and Methods:* A sample of 91 elderly females, aged between 60 and 81, were randomly divided into three groups (A, B, C). Each group performed the following order of exercises: Group A consisted of warm-up followed by aerobic training, strength training, stretching and cool down; Group B consisted of warm-up followed by strength training, aerobic training, stretching and cool down; while the control group (C) did not perform any exercise. Functional fitness and body composition were assessed at 3 moments of the 32 weeks (baseline and after each 16-week) intervention. One-way ANOVA for comparison between groups, ANOVA for repeated measures and multiple linear regression were used for statistical analysis. *Results:* The results showed that the functional fitness and body composition varied over the 32 weeks of multicomponent training. However, group A seems to show higher improvements in more variables. *Conclusion:* In the current study, group A obtained better results in most of the evaluated parameters. Thus, to improve functional fitness, warm-up, followed by aerobic training, strength training and relaxation may be the most suitable training for elderly women.

## 1. Introduction

In the XXI century, the number of elderly subjects has increased in developed countries [1]. The number of studies in this population, aiming to prevent ageing and the related morbidities have been increasing [2]. Ageing is related with different processes leading to a diminished capacity of adaptability and functional capacity [3,4]. Typically, ageing has been associated with higher levels of morbidity, functional incapacity, lower independency levels and higher mortality. The active lifestyles have been considered as determinant to delay the ageing negative effects. Upon that, physical activity, functional and fitness capacity has been considered as determinant to improve and maintain, for as long as possible, elders’ quality of life and autonomy [5,6].

The training programs based on strength and aerobic prescribed exercises, with adequate volume and frequency, may contribute to delay the motor and neuromuscular capacity decay [7,8]. Several authors have reported the positive effects of resistance training in strength levels and muscular mass, reducing sarcopenia [9,10,11,12,13]. Upon that, body composition changes due to age will also be minimized with physical exercise and activity [14,15]. Moreover, there are guidelines and recommendations to combine aerobics, strength, flexibility and balance exercises [16,17]. However, different outcomes are expected from different training and exercise types [14].

It is possible to identify in previous literature different types of training programs aiming to improve or preserve elderly’s physical fitness [14] and body composition [18]. However, the training sessions with varied approaches such as multimodal and multicomponent exercise sessions seemed to be related with more positive functional fitness outcomes [19,20,21]. The multicomponent training is characterized by aerobic, resistance, balance and flexibility exercises. This training type may improve cardiorespiratory fitness, metabolic outcomes, functional and cognitive performance [22]. Upon that, it is expected that the elderly’s quality of life may be improved with the multicomponent training [23,24]. The available literature reported multicomponent training interventions ranging between 9 and 48 weeks [24,25,26].

The literature presented one study assessing the effects of the order of the exercises in combined aerobic and resistance training on arterial stiffness in older men [27]. In obese men, the concurrent resistance-aerobic training type seemed to have higher effects on body composition [22]. Additionally, the studies showed that the sequence of the exercises influences the arterial stiffness and body composition [22,27]. To the authors’ best knowledge, no study was found assessing the effects of different order of exercises in a multicomponent training program, on elders’ body composition and functional fitness. Upon that, starting the multicomponent training sessions with aerobic-based exercises or resistance-based exercises after warm-up may have different effects on functional fitness and body composition. Typically, aerobic based exercises have been related to body fat reductions based on oxidative pathways, whereas resistance will induce a glycogenolysis approach firstly [22]. That may lead to different effects. Thus, the aim of this study was to evaluate the order of the exercise’s effects on body composition and functional fitness after 32 weeks. It was hypothesized that the order of the exercises may have different effects on body composition and physical fitness.

## 2. Materials and Methods

### 2.1. Sample

The sample of this study was composed of 91 elderly females, aged between 60 and 81 (69.62 ± 5.16 years), with body mass of 69.36 ± 10.53 kg and body mass index of 26.93 ± 4.07 kg/m^2^. The participants were randomly assigned into one of three groups (A, B or C). The volunteers were informed about the study aims and the protocol and were asked to sign an informed consent. All the procedures were in agreement with the Helsinki’s declaration. The research project received approval by the Scientific Board of the Higher Institute of Educational Sciences of the Douro (nº: 2.576). The participants were instructed to maintain normal levels of daily life activities to prevent physical inactivity. During the first visit the participants were asked to complete a sample characterization questionnaire. Table 1 describes the main characteristics of each group (A, B or C).

To participate in the present study, the inclusion criteria were as follows: (i) to be more than years old; (ii) independent in daily life activities; (iii) do not present chronic diseases with pharmacological medication that might affect the experimental protocol (such as cardiovascular, respiratory, metabolic syndrome or articular diseases). The exclusion criteria were as follows: (i) fail more than 25% of the total training sessions; (ii) to fail more than four consecutive sessions; and (iii) to fail the evaluation moments. The sampling was of convenience, where each participant was randomly assigned to 1 of the 3 groups, among which 2 intervention groups (*n*_A_ = 30; *n*_B_ = 32) and 1 control group (*n*_C_ = 29). Only the study sample was considered for analysis, and all drop outs were excluded from the database.

Generally, the participants were overweight, and there were no statistical differences between groups in terms of BMI or age characteristics at basal level (*p* > 0.05).

### 2.2. Multicomponent Training Program

The exercises combined aerobic, resistance, flexibility and balance exercises. This program was planned to consider the recommendations outlined by Carvalho et al. [23]. The sessions lasted between 50 and 60 min of 5 fundamental parts: (i) 5–8 min of warm-up, including slow walking and stretching exercises; (ii) walking involving aerobic exercises, jogging, aerobics and dancing (15–20 min), with a minimum of 8-10 min, and the intensity was maintained at 12–14 on the perceived exertion scale [28]; (iii) 1 to 3 sets of resistance exercises with elastic bands and free weights performed in a circuit (rest period of 40–60 s between sets), involving the main muscle groups such as knee flexors/extensors, shoulder abductors and adductors, flexors/extensors elbow, pectorals and abdominals. To allow a proper familiarization with the exercises and a correct execution of the breathing techniques, the training intensity was lower at the beginning of each month. Participants initially performed 8 reps in a single set and gradually progressed to 12 to 15 reps and 3 sets; (iv) static and dynamic balance training using sticks, balls and balloons for 5–8 min; and (v) at the end of each session, there was a cool-down period of about 5 min involving breathing exercises and stretching. The exercise program and the evaluations were applied by the researcher with knowledge of the methods, in the premises of the Escola Superior de Educação (ESE) do Instituto Politécnico de Bragança (IPB).

**Figure 1:**
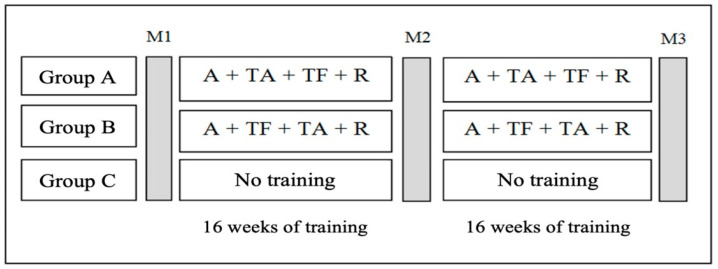
Illustrative study outline. Abbreviations: A—Warm-up; M1—first moment of evaluation, basal level; M2—second moment of evaluation; M3—third moment of evaluation, at the end of study; R—stretching and cool down; TA—Aerobic training; TF—strength training.

For the present study, two experimental groups had the same training methodology (multicomponent training). Each group participated in 3 morning (9:30 h–11:00 h) weekly session of the program taking 60 min each. However, the order of aerobic and strength exercises was changed in group B. Thus, in experimental group A, the order of exercises in the multicomponent training was warm-up followed by aerobic training, strength training, stretching and cool down; in experimental group B, the order of exercises was warm-up followed by strength training, aerobic training and relaxation. A control group (C) was also used where the participants did not have any physical exercise program. The Figure 1 represented the evaluated groups and training methodology.

Both groups were followed over 32 weeks and evaluated at three different moments: M1—first time of assessment or initial assessment, which coincided with the beginning of training; M2—second moment of evaluation or intermediate evaluation, after 16 weeks of training; M3—the third moment of assessment or final assessment, after 32 weeks of training. All the groups were performed the same tests to assess body composition and physical fitness.

### 2.3. Anthropometry and Body Composition

The lean mass, percentage of fat mass, bone mineral density, visceral fat, total body mass, muscle mass, fat mass and bone density were assessed wearing light clothing and without shoes using a digital bioimpedance scale (Tanita BC-50, Illinois, USA) before breakfast. The height was measured with subjects standing and head in the Frankfurt plane. BMI, expressed in kg/m^2^, was calculated using the formula (body mass/height^2^). The World Health Organization reference values for BMI were used (normal weight: between 18.50 and 24.99 body weight/height^2^; pre-obese: between 25 and 29.99 body weight/height^2^; Obese class I: between 30 and 34.99 body weight/height^2^; Obese class II: between 35 and 39.99 bodyweight/height^2^) [29].

### 2.4. Functional Fitness

Functional fitness was assessed using the Functional Fitness Test (FFT) [6], which was developed to assess the main physical parameters associated with functional mobility, consisting of 6 items: limb strength and endurance of lower (30-s chair stand and seat, by the number of repetitions) and upper limbs (arm curl with 2 kg dumbbell); lower flexibility (chair sit-and-reach, in centimeters) and superior flexibility (back scratch, in centimeters); physical mobility, speed, agility and dynamic balance (stand up the chair and run 8-ft up-and-go returning and seat on the chair, measured in seconds); and aerobic endurance (2 min step test, rising and counting the repetitions, when the knee reaches the hip level). This methodology has been used in previous studies [6,14,23].

### 2.5. Statistical Analysis

Descriptive analysis of the results was performed using the Statistical Package for the Social Sciences (SPSS) software, version 27.0 (IBM, Armonk, NY, USA). A *p* value of less than 0.05 was considered statistically significant. Continuous variables were expressed as means ± standard deviations. The distribution of data was tested using Shapiro–Wilk test or by analyzing the values of asymmetry and flatness. A One-way ANOVA was applied to compare between groups and a Repeated Measures ANOVA was applied to compare between moments. In case of violation of sphericity (TUG variable), the Greenhouse–Geisser correction was assumed. The post-hoc Bonferroni test was used for pairwise comparison. Δ1 was defined as the difference between M1 and M2, while Δ2 was defined as the difference between M1 and M3 [30]. Multiple linear regression analysis was performed using significant bivariates of variations in body composition and physical fitness, while controlling for the confounding factor: age [31].

The effect size is given by eta-squared (η^2^), the curvilinear ratio, that is, the ratio between the squared sum of the model and the total squared sum. The interpretation of the effect measure was made according to Ferguson [32]: if the effect measure > 0.640—high effect; if 0.250 < measure of effect ≤0.640—moderate effect; 0.040 < measurement of effect ≤0.250—reduced effect; and if 0 < measurement of effect ≤0.040—there is no effect.

## 3. Results

Table 2 shows the descriptive analysis for body composition and physical fitness of groups A, B and C during the intervention program. The descriptive statistics (mean values ± standard deviations) are described, along with report of statistically significant differences between groups and moments.

Regarding body composition, from the analysis of Table 2, the BMI comparisons between groups were statistically significant for delta 2 (F_(2,88)_ = 8.29; *p* < 0.001;$\;\eta^{2}$ = 0.16), with statistically significant differences between the control group and group A (*p* = 0.001) and the control group and group B (*p* = 0.003). Comparing between moments, there wasw a statistically significant decrease in group A (F_(2,58)_ = 11.13; *p* < 0.001;$\;\eta^{2}$ = 0.28), namely, between the first and the third moment (*p* = 0.002) and the second and third moment (*p* < 0.001). Group B also experienced a statistically significant decrease (F_(2,68)_ = 10.11; *p* < 0.001;$\;\eta^{2}$ = 0.25), namely, between the first and third moments (*p* = 0.016) and the second and third moments (*p* < 0.001).

The basal metabolisms (BM) were statistically significant different between groups in all the measurements, with the exception of delta 1. For M1 (F_(2,88)_ = 8.66; *p* < 0.001;$\;\eta^{2}$ = 0.17), there were statistically significant differences between the control group and group A (*p* = 0.040) and between group A and B (*p* < 0.001). For M2 (F_(2,88)_ = 10.06; *p* < 0.001;$\;\eta^{2}$ = 0.19), there were statistically significant differences between the control group and group A (*p* = 0.023) and between group A and B (*p* < 0.001). For M3 (F_(2,88)_ = 8.95; *p* < 0.001;$\;\eta^{2}$ = 0.17) there were statistically significant differences between the control group and group A (*p* = 0.006) and between group A and B (*p* < 0.001). Delta 2 was statistically different between groups (F_(2,88)_ = 8.54; *p* < 0.001;$\;\eta^{2}$ = 0.16), and there were statistically significant differences between the control group and group A (*p* = 0.008) and between the control group and group B (*p* < 0.001). When comparing between moments, there was a significant increase in group A (F_(2,58)_ = 4.97; *p* = 0.010;$\;\eta^{2}$ = 0.15), namely, between the second and the third moment (*p* = 0.031). Group B also experienced a significant statistical increase (F_(2,62)_ = 14.26; *p* < 0.001;$\;\eta^{2}$ = 0.32), namely, between the first and third moments (*p* < 0.001) and the second and third moments (*p* < 0.001).

Water (W) was statistically significant different between groups only in the third moment of evaluation and delta 2. For M3 (F_(2,88)_ = 7.69; *p* < 0.001;$\;\eta^{2}$ = 0.15), there were statistically significant differences between the control group and group A (*p* < 0.001) and between the control group and group B (*p* = 0.042). For delta 2 (F_(2,88)_ = 30.29; *p* < 0.001;$\;\eta^{2}\;$= 0.41), there were statistically significant differences between the control group and group A (*p* < 0.001) and between the control group and group B (*p* < 0.001). When comparing between moments of evaluation, there was a significant decrease in the control group (F_(2,56)_ = 13.67; *p* < 0.001;$\;\eta^{2}$ = 0.33), namely, between the first and second measurement (*p* = 0.006) and the first and the third measurement (*p* < 0.001). Group A increased statistically significantly between measurements (F_(2,58)_ = 14.43; *p* < 0.001;$\;\eta^{2}$ = 0.33), namely, between the first and third measurement (*p* < 0.001) and the second and the third measurement (*p* < 0.001). Group B increased statistically significantly between measurements (F_(2,62)_ = 20.92; *p* < 0.001;$\;\eta^{2}\;$= 0.41), namely, between the first and third measurement (*p* < 0.001) and the second and the third measurement (*p* < 0.001).

Visceral Fat (VF) was statistically different between groups, for all the moments and for delta 2. For M1 (F_(2,88)_ = 3.86; *p* = 0.025;$\;\eta^{2}$ = 0.08), there were statistically significant differences between group A and group B (*p* = 0.023). For M2 (F_(2,88)_ = 4.04; *p* = 0.021;$\;\eta^{2}$ = 0.08), there were statistically significant differences between group A and group B (*p* = 0.018). For M3 (F_(2,88)_ = 3.86; *p* = 0.039;$\;\eta^{2}$ = 0.07), there were statistically significant differences between group A and group B (*p* = 0.037). Delta 2 (F_(2,88)_ = 4.19; *p* = 0.018;$\;\eta^{2}$ = 0.09), had statistically significant differences between the control group and group A (*p* = 0.018). For between moments comparison, visceral fat decreased during the program for both group A and B. Group A (F_(2,58)_ = 4.35; *p* = 0.017;$\;\eta^{2}$ = 0.13), decreased significantly between the second and third moment (*p* = 0.018), while group B (F_(2,62)_ = 10.42; *p* < 0.001;$\;\eta^{2}$ = 0.25), decreased significantly from the first to the third moment (M1 vs. M3 *p* = 0.002; M2 vs. M3 *p* = 0.002).

Bone mass (BOM) was statistically different between group A and group B for all the moments (M1: (F_(2,88)_ = 6.22; *p* = 0.003;$\;\eta^{2}$ = 0.12; A/B *p* = 0.002); M2 (F_(2,88)_ = 7.08; *p* = 0.001;$\;\eta^{2}$ = 0.14; A/B *p* < 0.001); M3 (F_(2,88)_ = 6.75; *p* = 0.002;$\;\eta^{2}$ = 0.13; Control/A *p* = 0.045 e A/B *p* = 0.002)). Delta 2 was statistically different between control and group B (F(_2,88)_ = 4.31; *p* = 0.016;$\;\eta^{2}$ = 0.09; C/B *p* = 0.019). The between moments comparison suggests an increase in bone mass for group A and group B. Group A (F_(2,58)_ = 8.60; *p* < 0.001;$\;\eta^{2}$ = 0.23) had statistical significant differences between the first and third measurement (*p* = 0.007) and the second and third measurements (*p* = 0.005). Group B (F_(2, 62)_ = 5.20; *p* = 0.008;$\;\eta^{2}$ = 0.14) had a statistically significant increase between the first and the third measurement (*p* = 0.031) and the second and the third measurement (*p* = 0.009).

Lean mass was consistently statistically different between group A and B for all the moments; however, the measure of variation (represented by deltas) did not differ significantly between groups. For M1 (F_(2,88)_ = 6.66; *p* = 0.002;$\;\eta^{2}$ = 0.13), there were statistically significant differences between group A and group B (*p* = 0.001). For M2 (F_(2,88)_ = 6.94; *p* = 0.002;$\;\eta^{2}$ = 0.14), there were statistically significant differences between group A and group B (*p* = 0.001). For M3 (F_(2,88)_ = 5.42; *p* = 0.006;$\;\eta^{2}$ = 0.11), there were statistically significant differences between group A and group B (*p* = 0.007). Between moments comparison showed a decrease in lean mass in the control group, while group A did not differ significantly between moments, and in group B, there was an increase in lean mass. Control group (F_(2,56)_ = 5.57; *p* = 0.006;$\;\eta^{2}$ = 0.17) demonstrated a significant decrease between the first and second measurement (*p* = 0.003) and the first and third measurement (0.042). Group B (F_(2,62)_ = 3.96; *p* = 0.024;$\;\eta^{2}$ = 0.11) exhibited a significant increase between the first and third measurement (*p* = 0.047).

Fat mass did not differ significantly between groups during the first and second measurements nor for delta 1, but at the third moment, there was a significant difference between the control group and group A (F_(2,88)_ = 4.56; *p* = 0.013;$\;\eta^{2}$ = 0.09, control group vs. group A *p* = 0.010). The variation in fat mass between the first and third measurement (Delta 2) differed significantly between the control group and both the test groups (F_(2,88)_ = 7.11; *p* = 0.001;$\;\eta^{2}$ = 0.14), demonstrating a positive delta for the control group, while the test groups demonstrated a negative variation (pairwise comparison between the control group and group A *p* = 0.004 and control group and group B *p* = 0.005). Between moments comparison demonstrated an increase in fat mass in the control group between the first and the third moment (*p* = 0.017; (F_(2,56)_ = 5.88; *p* = 0.005;$\;\eta^{2}$ = 0.17)). Group A evidenced a decrease in fat mass between the second and third measurements (*p* = 0.035; (F_(2,58)_ = 4.90; *p* = 0.011;$\;\eta^{2}$ = 0.15). Group B showed a significant decrease between the first and the third measurements (p = 0.002) and the second and third measurement (*p* = 0.014); (F_(2,62)_ = 10.17; *p* < 0.001;$\;\eta^{2}$ = 0.25).

Regarding Physical fitness variables, the arm curl (AC) comparisons between groups were statistically significant for the second and third moment of evaluation (M1 and M2), and for delta 2. At the second moment of evaluation (F_(2,88)_ = 3.21; *p* = 0.045;$\;\eta^{2}$ = 0.07), there were statistically significant differences between control group and group A (*p* = 0.040) and control group and group B (*p* = 0.023). The third moment of evaluation (F_(2,88)_ = 3.75; *p* = 0.027;$\;\eta^{2}$ = 0.08) showed statistically significant differences between the control group and group A (*p* = 0.045). Delta 2 (F_(2,88)_ = 5.08; *p* = 0.008;$\;\eta^{2}$ = 0.10) demonstrated statistically significant differences between the control group and group A (*p* = 0.007). Comparing between moments, there was a significant decrease in arm curls for the control group (F_(2,56)_ = 10.56; *p* < 0.001;$\;\eta^{2}$ = 0.28), namely, between the first and the second moment (*p* = 0.009), the first and the third moment (*p* = 0.005) and the second and third moment (*p* = 0.041), while group A and B did not show differences between moments.

The back scratch (BS) test did not differ between groups for any of the moments, with exception for the variation between the first and the third moments (delta 2) that was statistically significant (F_(2,88)_ = 6.71; *p* = 0.002;$\;\eta^{2}$ = 0.13), with statistically significant differences between the control group and group A (*p* = 0.002) and the control group and group B (*p* = 0.035). Comparing between moments, there was a significant increase in back scratch (BS) for the control group (F_(2,56)_ = 4.82; *p* = 0.012;$\;\eta^{2}$ = 0.15), namely, between the second and the third moments (*p* = 0.040). Group A showed a consistent decrease (F_(2,58)_ = 11.22; *p* < 0.001;$\;\eta^{2}$ = 0.28) for delta 2 (*p* = 0.004) and delta 1 (*p* < 0.001). Group B showed a decrease (F_(2,62)_ = 4.06; *p* = 0.022;$\;\eta^{2}$ = 0.12) for delta 1 (*p* = 0.006).

Sit and reach (SR) demonstrated statistically significant differences between the control group and group B for all moments of evaluation, with the exception of delta 1, which did not differ between groups. Moreover, delta 2 showed differences between the control group and both group A and B. For detailed information: For the first moment of evaluation (F_(2,88)_ = 3.83; *p* = 0.025;$\;\eta^{2}$ = 0.08), there were statistically significant differences between the control group and group B (*p* = 0.026). For the second moment of evaluation (F_(2,88)_ = 5.58; *p* = 0.005;$\;\eta^{2}$ = 0.11), there were statistically significant differences between the control group and group B (*p* = 0.004). For the third moment of evaluation (F_(2,88)_ = 6.86; *p* = 0.002;$\;\eta^{2}$ = 0.14), there were statistically significant differences between the control group and group B (*p* = 0.001). For delta 2 (M3-M1) (F_(2,88)_ = 4.73; *p* = 0.01;$\;\eta^{2}$ = 0.1), there were statistically significant differences between the control group and group A (*p* = 0.019) and the control group and group B (*p* = 0.040). Comparing between moments, there was no difference throughout the study for the control group; however, a significant increase in sit and reach (SR) was observed both for group A and B. Group A (F_(2,58)_ = 6.72; *p* = 0.002;$\;\eta^{2}$ = 0.19) showed differences between the first and the third moments (*p* = 0.004), while group B (F_(2,62)_ = 4.34; p = 0.017;$\;\eta^{2}$ = 0.12) also showed differences between the first and third measurements (*p* = 0.024).

Differences between groups for Time up and go (TUG) showed statistical significance for all moments of evaluation; however, both measures of variation (deltas) did not show differences between groups. For the first moment of evaluation (F_(2,88)_ = 5.70; *p* = 0.005;$\;\eta^{2}$ = 0.12), there were statistically significant differences between the control group and group A (*p* = 0.004). The second moment of evaluation (F_(2,88)_ = 8.23; *p* < 0.001;$\;\eta^{2}$ = 0.16) had statistically significant differences between the control group and group A (*p* < 0.001) and the control group and group B (*p* = 0.011). The third moment of evaluation (F_(2,88)_ = 8.75; *p* < 0.001;$\;\eta^{2}$ = 0.17) had statistically significant differences between the control group and group A (*p* < 0.001) and the control group and group B (*p* = 0.005). Comparing between moments, there was no difference throughout the study for the control group; however, a significant decrease in TUG was observed both for group A and B. Group A (F(_2,58)_ = 11.41; *p* < 0.001;$\;\eta^{2}$ = 0.28) showed differences between the first and the third moments (*p* = 0.001) and the second and third moments (*p* = 0.004). Group B (F_(2,62)_ = 8.86; *p* < 0.001;$\;\eta^{2}$ = 0.22) also showed differences between the first and third measurements (*p* = 0.003) and the second and third moments (*p* = 0.002).

The chair stand test (CS) only showed differences between groups at the third moment of evaluation and for delta 2 (the variation between the third and first moments), demonstrating differences between the control group and both groups A and B. The third moment of evaluation (F_(2,88)_ = 7.03; *p* < 0.001;$\;\eta^{2}$ = 0.14) had statistically significant differences between the control group and group A (*p* = 0.015) and the control group and group B (*p* = 0.002). Delta 2 (F_(2,88)_ = 13.52; *p* < 0.001;$\;\eta^{2}$ = 0.24) had statistically significant differences between control group and group A (*p* < 0.001) and the control group and group B (*p* < 0.001). Comparing between moments, there was a consistent decrease in the value of CS for the control group, while group A and B experienced a consistent increase in the variable. The control group (F_(2,56)_ = 9.28; *p* < 0.001;$\;\eta^{2}$ = 0.25) demonstrated a significant decrease between the first and third measurements (*p* = 0.002) and the second and third measurements (*p* = 0.033). Group A (F_(2,58)_ = 8.41; *p* < 0.001;$\;\eta^{2}$ = 0.23) showed differences between the first and the third moments (*p* = 0.008) and the second and third moments (*p* = 0.015). Group B (F_(2,62)_ = 17.54; *p* < 0.001;$\;\eta^{2}$ = 0.36) also showed differences between the first and third measurements (*p* < 0.001) and the second and third measurements (*p* = 0.011).

The two-minute step test (2ST) demonstrated statistically significant differences between the groups for all the moments and both deltas, with the exception of the third moment of evaluation (M3) that did not show differences between groups. Specifically, M1 (F_(2,88)_ = 12.43; *p* < 0.001;$\;\eta^{2}$ = 0.22) had statistically significant differences between the control group and group A (*p* < 0.001) and the control group and group B (*p* < 0.001). M2 (F_(2,88)_ = 3.60; *p* = 0.031;$\;\eta^{2}$ = 0.08) had statistically significant differences between the control group and group A (*p* = 0.020) and the control group and group B (p = 0.024). Delta 1 (F_(2,88)_ = 5.34; *p* = 0.006;$\;\eta^{2}$ = 0.11) had statistically significant differences between the control group and group B (*p* = 0.005). Delta 2 (F_(2,88)_ = 16.41; *p* < 0.001;$\;\eta^{2}$ = 0.27) had statistically significant differences between the control group and group A (*p* < 0.001) and the control group and group B (*p* < 0.001). When comparing between moments, there was a consistent decrease in the two-minute step test for the control group, while group A and B experienced a consistent increase in the variable. The control group (F_(2,56)_ = 17.62; *p* < 0.001;$\;\eta^{2}$ = 0.39) demonstrated a significant decrease between the first and third measurements (*p* < 0.001) and the second and third measurements (*p* < 0.001). Group A (F_(2,58)_ = 5.20; *p* = 0.008;$\;\eta^{2}$ = 0.15) showed differences between the first and the third moments (*p* = 0.006). Group B (F_(2,62)_ = 9.11; *p* < 0.001;$\;\eta^{2}$ = 0.23) also showed differences between the first and second measurements (*p* = 0.002) and the first and third measurements (*p* < 0.001).

The following table (Table 3) expresses the results for the multiple regression analysis for the variations identified as significant in the previous analysis, while controlling for the confounding factor: age.

From the analysis of Table 3, it is suggested that the R2 statistics ranged between 7% (LM) and 41% (W) of the total variance explained. The adjusted R2 ranged between 3% and 39%, which is indicative of the effect size. All the reported models demonstrated significance, with exception of LM. Being part of group A represented a bigger slope in 7 (BMI, W, VF, FM, AC, BS, SR) out of 12 significant models. On the other hand, being part of group B represented a bigger slope in 5 (BM, BOM, CS and both deltas for 2ST) out of 12 significant models. All the significant slopes are indicative of a significant difference between the referred group and the control group in terms of variation between the first and the third moment (delta 2) or the first and the second moment (delta 1).

## 4. Discussion

The main purpose of this study was to verify the effects of a Multicomponent training program on body composition, isometric strength, and functional fitness over 32 weeks. As such, the sample was divided into three groups, a control group (group C) and two experimental groups (group A and group B), to which the order of aerobic training and strength training was reversed.

No significant differences were found in BMI for all groups. However, high values were observed. These are in line with the literature that explains an increasing tendency in total fat mass and a decreasing in muscle mass due to the aging process [23,33]. Leite et al. [34] reported that near 70 years old, the elderly have between 25% to 40% of fat mass, mostly explained by the aging process. Other important changes occur such as the loss of bone mineral mass, a decrease in resting metabolic rate and a substantial decrease in lean mass or fat-free mass (10 a 16%, due to losses in bone mass, skeletal muscle, and total body water) [35]. The practice of regular multivariate physical exercise programs, such as the one used in this study, had beneficial effects on body composition, increasing lean mass and decreasing the percentage of fat mass [34,36,37].

In the present study and considering the assessment of body composition by groups over the three assessment moments, it is possible to verify that both group A and group B decreased BMI, visceral fat and fat mass. These results are in accordance with the literature [38] on the effects of concurrent training, verifying a reduction in the percentage of fat mass between baseline and after the training program. Additionally, there were increases in the basal metabolism, water, bone mass and lean mass. This is frequent after physical exercise programs, where the effects remain for a long period of time [39]. In group C (no physical exercise), there was an inverse trend: increases in BMI, visceral fat, fat mass and decreases in basal metabolism, water, mineral bone mass and lean mass.

During the training program and at different evaluations, both groups (A and B) obtained significant gains in the back scratch variables; sit and reach; chair stand; 2-min step test; the time up and go variable had a significant decrease meaning better agility results. The results of the present study are in agreement with other research studies, presenting improvements in the chair stand and sit and reach test, pointing out the responsibility of such performance to the multicomponent training programs [39,40]. For the group’s comparisons, it was possible to find significant differences between groups in the sit and reach variables for groups B and C; in the time up and go between A and C and between B and C; for the chair stand between C and A and between C and B.

Literature provides support about the effects of multicomponent training in the elderly. Exercise and physical activity are related to high levels of functional fitness, and the smallest change may be extremely important to the maintenance of functionality in daily tasks, as well as for the prevention of falls [13,41]. Regarding resistance training in multicomponent programs, it is verified that such strength gains revealed improvements in the ability to stand up and sit from a chair [9,10] or lean forward, allowing the elderly the ability to continue with their daily activities [42]. Regarding the aerobic endurance, the decrease in aerobic endurance is age-related and influences daily life activities [43]. Moreover, the multicomponent training appears in the literature associated with improvements in aerobic endurance performance in the elderly [44]. The results of the present study may be supported or not [44,45]. Combining strength and endurance training in the elderly has been described as a valid strategy to improve neuromuscular and cardiorespiratory function during aging [44]. However, the influence of intra-session sequence is not corroborated in previous research [45]. This may be explained by the lack of specificity of the multicomponent training and the sample characteristics. Thus, more studies are required to better understand the effects of the order of the exercises.

The multiple regression results indicated that group A presented a higher slope for most of the variables between the first and last moment. This supports the fact that group A had higher improvement in comparison to group B. Such evidence recommends multicomponent training combining strength training and aerobic training as the best strategy to maintain functional capacity during aging [38,41,44]. The test sequence seems to influence strength and endurance gains, being optimized if strength training is performed first [44]. The present study refutes this trend, and it was found that group A with the sequence of exercises: warm-up followed by aerobic training, strength training, and relaxation presented higher values for the elderly population that intends to increase muscle strength and functional fitness, contrary to group B with the sequence of exercises warm-up followed by strength training, aerobic training and relaxation.

The present study has some limitations that may influence the extrapolation of the results, such as the absence of a dietary control, which did not allow for a more critical analysis of the effect of training and diet on body composition. Another limitation is due to the daily routines, since the daily physical activity was not controlled, making it impossible to have a more critical analysis of the training effect. Finally, the sample of this study has very specific characteristics, such as not being under any pharmacological treatment and the absence of chronic diseases, which is less probable in this range of ages. The VO_2_ maximal capacity was not assessed or estimated and the use of bioimpedance to assess body composition are presented as limitations of the study. However, it is important to highlight the results of the present study as they provide indications about the efficiency of training in elderly women, suggesting that independent elderly women should seek physical exercise programs to improve or maintain their functional performance, ensuring the autonomy and independence during aging.

## 5. Conclusions

The results showed that group A obtained better results in most of the evaluated parameters, which allows us to conclude that the multicomponent training consisting of the exercise sequence warm-up, followed by aerobic training, strength training and relaxation, is the most suitable training for the elderly population who want to increase muscle strength and functional fitness. Upon that, the group A may allow to better improve elderly people’s quality of life, instead of the training order of group B.

## Figures and Tables

**Table 1:** Descriptive statistics for Group composition, age and BMI.

Group—Participation	*n*	Age (Mean ± SD)	BMI (Mean ± SD)
A—Intervention	30	69.40 ± 5.24	27.04 ± 2.87
B—Intervention	32	70.63 ± 5.15	26.34 ± 3.95
C—Control	29	68.72.84 ± 5.09	27.46 ± 5.16
Significance (*p* value)	--	0.347	0.558

**Table 2:** Description of body composition, physical fitness in each group in the three moments of the program.

	Variables		Control	Group A	Group B	*p* Value Group Compare	Effect Size η^2^
**Body composition**	**BMI (Kg/m^2^)**	M1 (Mean ± sd)	27.46 ± 5.16	27.04 ± 2.87	26.34 ± 3.95	0.558 (n.s.)	0.01
		M2 (Mean ± sd)	27.57 ± 5.11	27.03 ± 3.00	26.35 ± 3.98	0.510 (n.s.)	0.02
		M3 (Mean ± sd)	27.71 ± 5.31	26.69 ± 2.79	26.06 ± 3.98	0.296 (n.s.)	0.03
		Δ1 (M2-M1)	0.11 ± 0.06	− 0.00 ± 0.08	0.01 ± 0.07	0.453 (n.s.)	0.02
		Δ2 (M3-M1)	0.25 ± 0.14	−0.35 ± 0.09 ± *	−0.29 ± 0.10 ± *	<0.001 †‡	0.16
		*p* Repeat Meas	0.146 (n.s.)	<0.001	<0.001		
		Effect Size	0.07	0.28	0.25		
	**BM (Kcal)**	M1 (Mean ± sd)	1348.28 ± 191.27	1468.93 ± 229.35	1273.91 ± 115.43	<0.001 †♦	0.17
		M2 (Mean ± sd)	1342.86 ± 197.13	1474.07 ± 221.16	1265.72 ± 124.83	<0.001 †♦	0.19
		M3 (Mean ± sd)	1340.45 ± 192.41	1491.53 ± 221.33	1304.19 ± 127.20	<0.001 †♦	0.17
		Δ1 (M2-M1)	−5.41 ± 4.74	5.13 ± 6.80	− 8.19 ± 8.19	0.351 (n.s.)	0.02
		Δ2 (M3-M1)	−7.83 ± 5.07	22.60 ± 9.10	30.28 ± 5.69 ± *	<0.001 †‡	0.16
		*p* Repeat Meas	0.377 (n.s.)	0.010	<0.001		
		Effect Size	0.03	0.15	0.32		
	**W (%)**	M1 (Mean ± sd)	46.88 ± 4.55	48.85 ± 5.46	47.45 ± 3.27	0.224 (n.s.)	0.03
		M2 (Mean ± sd)	46.17 ± 4.74	49.08 ± 5.36	47.38 ± 3.50	0.054 (n.s.)	0.06
		M3 (Mean ± sd)	45.69 ± 4.96	50.45 ± 5.63	48.72 ± 3.37	<0.001 †‡	0.15
		Δ1 (M2-M1)	−0.70 ± 0.21 ± *	0.23 ± 0.33	−0.08 ± 0.25	0.051(n.s.)	0.065
		Δ2 (M3-M1)	−1.19 ± 0.22 ± *	1.60 ± 0.37 ± *	1.27 ± 0.21 ± *	<0.001 †‡	0.41
		*p* Repeat Meas	<0.001	<0.001	<0.001		
		Effect Size	0.33	0.33	0.40		
	**VF (%)**	M1 (Mean ± sd)	9.93 ± 3.60	11.47 ± 3.20	9.19 ± 3.04	0.025♦	0.08
		M2 (Mean ± sd)	10.00 ± 3.60	11.53 ± 3.20	9.19 ± 3.06	0.021♦	0.08
		M3 (Mean ± sd)	10.10 ± 3.75	10.83 ± 2.95	8.78 ± 2.77	0.039♦	0.07
		Δ1 (M2-M1)	0.069 ± 0.048	0.07 ± 0.23	0.00 ± 0.09	0.927(n.s.)	0.01
		Δ2 (M3-M1)	0.172 ± 0.112	−0.63 ± 0.31	−0.41 ± 0.11 ± *	0.018 †	0.09
		*p* Repeat Meas	0.179 (n.s.)	0.017	<0.001		
		Effect Size	0.06	0.13	0.25		
	**BOM (Kg)**	M1 (Mean ± sd)	2.30 ± 0.38	2.45 ± 0.41	2.14 ± 0.25	0.003♦	0.12
		M2 (Mean ± sd)	2.30 ± 0.38	2.46 ± 0.38	2.15 ± 0.19	0.001♦	0.14
		M3 (Mean ± sd)	2.30 ± 0.37	2.51 ± 0.38	2.21 ± 0.20	0.002 †♦	0.13
		Δ1 (M2-M1)	−0.00 ± 0.00	0.01 ± 0.01	0.01 ± 0.03	0.837 (n.s.)	0.01
		Δ2 (M3-M1)	−0.00 ± 0.01	0.06 ± 0.02 ± *	0.07 ± 0.03 ± *	0.016 ‡	0.09
		*p* Repeat Meas	0.785 (n.s.)	<0.001	0.008		
		Effect Size	0.01	0.23	0.14		
	**LM (Kg)**	M1 (Mean ± sd)	43.08 ± 7.53	46.39 ± 7.76	40.31 ± 3.81	0.002♦	0.13
		M2 (Mean ± sd)	42.94 ± 7.50	46.45 ± 7.54	40.35 ± 3.78	0.002♦	0.14
		M3 (Mean ± sd)	42.87 ± 7.55	47.06 ± 7.65	41.66 ± 4.73	0.006♦	0.11
		Δ1 (M2-M1)	−0.14 ± 0.04 ± *	0.06 ± 0.26	0.04 ± 0.12	0.643 (n.s.)	0.01
		Δ2 (M3-M1)	−0.22 ± 0.08 ± *	0.67 ± 0.35	1.35 ± 0.65 ± *	0.051 (n.s.)	0.07
		*p* Repeat Meas	0.006	0.05 (n.s.)	0.024		
		Effect Size	0.17	0.10	0.11		
	**FM (%)**	M1 (Mean ± sd)	35.71 ± 5.91	32.26 ± 7.86	34.64 ± 5.86	0.124 (n.s.)	0.05
		M2 (Mean ± sd)	35.83 ± 5.79	31.99 ± 8.06	34.47 ± 5.70	0.082 (n.s.)	0.06
		M3 (Mean ± sd)	36.17 ± 5.95	30.92 ± 8.40	33.36 ± 5.37	0.013 †	0.09
		Δ1 (M2-M1)	0.11 ± 0.11	−0.27 ± 0.42	−0.18 ± 0.19	0.594 (n.s.)	0.01
		Δ2 (M3-M1)	0.46 ± 0.15 ± *	−1.34 ± 0.53	−1.28 ± 0.34 ± *	0.001 †‡	0.14
		*p* Repeat Meas	0.005	0.011	<0.001		
		Effect Size	0.17	0.15	0.25		
**AC (Rep)**	M1 (Mean ± sd)	23.07 ± 5.04	24.07 ± 5.30	24.53 ± 4.76	0.517 (n.s.)	0.02
**Physical fitness**		M2 (Mean ± sd)	22.66 ± 4.85	25.43 ± 6.09	25.69 ± 4.29	0.045 †‡	0.07
		M3 (Mean ± sd)	22.28 ± 5.06	25.70 ± 6.36	25.37 ± 4.35	0.027 †	0.08
		Δ1 (M2-M1)	−0.41 ± 0.13 ± *	1.37 ± 0.62	1.16 ± 0.72	0.063 (n.s.)	0.06
		Δ2 (M3-M1)	−0.79 ± 0.23 ± *	1.63 ± 0.69	0.84 ± 0.57	0.008 †	0.10
		*p* Repeat Meas	<0.001	0.058 (n.s.)	0.213 (n.s.)		
		Effect Size	0.27	0.09	0.05		
	**BS (cm)**	M1 (Mean ± sd)	−8.78 ± 10.26	−9.05 ± 7.91	−5.53 ± 6.35	0.180 (n.s.)	0.04
		M2 (Mean ± sd)	−8.72 ± 10.42	−8.97 ± 8.20	−5.59 ± 6.32	0.216 (n.s.)	0.03
		M3 (Mean ± sd)	−9.34 ± 10.87	−7.50 ± 7.64	−4.59 ± 6.54	0.092 (n.s.)	0.05
		Δ1 (M2-M1)	0.05 ± 0.13	0.08 ± 0.31	−0.06 ± 0.37	0.933 (n.s.)	0.00
		Δ2 (M3-M1)	−0.057 ± 0.28	1.55 ± 0.43 ± *	0.94 ± 0.49	0.002 †‡	0.13
		*p* Repeat Meas	0.012	<0.001	0.022		
		Effect Size	0.15	0.28	0.12		
	**SR (Rep)**	M1 (Mean ± sd)	−1.90 ± 11.30	0.27 ± 11.32	5.11 ± 7.65	0.025 ‡	0.08
		M2 (Mean ± sd)	−1.92 ± 11.46	1.50 ± 9.79	6.25 ± 7.33	0.005 ‡	0.11
		M3 (Mean ± sd)	−1.92 ± 11.35	2.43 ± 9.51	7.03 ± 7.22	0.002 ‡	0.14
		Δ1 (M2-M1)	−0.02 ± 0.10	1.23 ± 0.70	1.14 ± 0.55	0.18 (n.s.)	0.04
		Δ2 (M3-M1)	−0.03 ± 0.12	2.17 ± 0.61 ± *	1.92 ± 0.68 ± *	0.01 †‡	0.10
		*p* Repeat Meas	0.96 (n.s.)	0.002	0.017		
		Effect Size	0.00	0.19	0.12		
	**TUG (Seg)**	M1 (Mean ± sd)	5.07 ± 0.79	4.40 ± 0.71	4.61 ± 0.81	0.005 †	0.12
		M2 (Mean ± sd)	5.07 ± 0.79	4.28 ± 0.65	4.48 ± 0.88	<0.001 †‡	0.16
		M3 (Mean ± sd)	4.93 ± 1.16	4.00 ± 0.65	4.17 ± 0.87	<0.001 †‡	0.17
		Δ1 (M2-M1)	0.01 ± 0.01	−0.12 ± 0.08	−0.14 ± 0.11	0.418 (n.s.)	0.02
		Δ2 (M3-M1)	−0.13 ± 0.17	−0.41 ± 0.10 *	−0.44 ± 0.12 *	0.213 (n.s.)	0.04
		*p* Repeat Meas	0.424 (n.s.)	<0.001	<0.001		
		Effect Size	0.02	0.28	0.22		
	**CS (cm)**	M1 (Mean ± sd)	20.38 ± 5.55	20.33 ± 4.77	20.84 ± 5.50	0.915 (n.s.)	0.00
		M2 (Mean ± sd)	20.21 ± 5.70	21.60 ± 5.12	22.50 ± 5.54	0.263 (n.s.)	0.03
		M3 (Mean ± sd)	19.66 ± 5.56	23.77 ± 5.05	24.66 ± 5.83	<0.001 †‡	0.14
		Δ1 (M2-M1)	−0.17 ± 0.12	1.27 ± 0.74	1.66 ± 0.73	0.097 (n.s.)	0.05
		Δ2 (M3-M1)	−0.72 ± 0.19 *	3.43 ± 1.05 ± *	3.81 ± 0.50 *	<0.001 †‡	0.24
		*p* Repeat Meas	<0.001	<0.001	<0.001		
		Effect Size	0.25	0.23	0.36		
	**2 ST (Rep)**	M1 (Mean ± sd)	117.55 ± 16.98	99.33 ± 20.50	95.63 ± 16.89	<0.001 †‡	0.22
		M2 (Mean ± sd)	116.10 ± 17.81	104.87 ± 20.96	105.38 ± 15.56	0.031 †‡	0.08
		M3 (Mean ± sd)	112.31 ± 17.41	108.90 ± 22.07	106.13 ± 17.60	0.454 (n.s.)	0.02
		Δ1 (M2-M1)	−1.45 ± 0.88	5.53 ± 3.09	9.74 ± 2.66 *	0.006 ‡	0.11
		Δ2 (M3-M1)	−5.24 ± 1.10 *	9.57 ± 2.82 ± *	10.50 ± 2.13 *	<0.001 †‡	0.27
		*p* Repeat Meas	<0.001	0.008	<0.001		
		Effect Size	0.39	0.15	0.23		

* represents *p* < 0,05 for delta based on ANOVA for repeated measures; † represents *p* < 0.05 for comparison between group Control and Group A; ‡ represents *p* < 0.05 for comparison between group Control and Group B; ♦ represents *p* < 0.05 for comparison between group A and Group B. Abbreviations: BMI—Body Mass Index; BM—Basal metabolism; W—Water; VF—Visceral fat; BOM—Bone mass; LM—Lean mass; FM—Fat mass; AC—Arm curl; BS—Back scratch; SR—Sit and reach; TUG—Time Up and Go; CS—Chair stand; 2 ST—2 min step test; M1—first moment of evaluation; basal level, M2—second moment of evaluation; M3—third moment of evaluation, at the end of study.

**Table 3:** Multiple regression results for the significant variations between the first and third moments of evaluation (Δ2) and between the first and second moments of evaluation (Δ1), adjusted for the confounding factor “age”.

	Variables		*B*	95% CI for B		SE B	β	R^2^	ΔR^2^	F	Sig.
				LL	UL						
**Body composition**	**BMI (Kg/m^2^)**	Δ2 Model						0.18	0.15	6.43	<0.001
		Constant	1.60	−0.14	3.33	0.87					
		Group A	−0.58 *	−0.89	−0.26	0.16	−0.41 *				
		Group B	−0.49 *	−0.81	−0.18	0.16	−0.36 *				
		Age	−0.02	−0.05	0.01	0.01	−0.15				
	**BM (Kcal)**	Δ2 Model						0.17	0.14	5.91	0.001
		Constant	−53.08	−160.56	54.40	54.08					
		Group A	29.98 *	10.41	49.56	9.85	0.35 *				
		Group B	36.86 *	17.39	56.32	9.79	0.43 *				
		Age	0.66	−0.89	2.21	0.78	0.08				
	**W (%)**	Δ2 Model						0.41	0.39	20.04	<0.001
		Constant	−1.98	−6.28	2.32	2.16					
		Group A	2.78	2.00	3.56	0.39	0.68 *				
		Group B	2.43	1.65	3.21	0.39	0.61 *				
		Age	0.01	−0.05	0.07	0.03	0.03				
	**VF (%)**	Δ2 Model						0.10	0.06	3.04	0.033
		Constant	1.55	−1.59	4.69	1.58					
		Group A	−0.79 *	−1.36	−0.22	0.29	−0.33 *				
		Group B	−0.54	−1.11	0.03	0.29	−0.23				
		Age	−0.02	−0.07	0.03	0.02	−0.09				
	**BOM (Kg)**	Δ2 Model						0.09	0.06	2.96	0.037
		Constant	−0.09	−0.39	0.21	0.15					
		Group A	0.06 *	0.01	0.11	0.03	0.26 *				
		Group B	0.07 *	0.02	0.13	0.03	0.32 *				
		Age	0.00	−0.00	0.01	0.00	0.06				
	**LM (Kg)**	Δ2 Model						0.07	0.03	2.03	0.115
		Constant	−0.55	−7.60	6.50	3.55					
		Group A	0.89	−0.40	2.17	0.65	0.17				
		Group B	1.56 *	0.28	2.83	0.64	0.30 *				
		Age	0.01	−0.10	0.11	0.05	0.01				
	**FM (%)**	Δ2 Model						0.14	0.11	4.74	0.004
		Constant	1.58	−4.38	7.55	3.00					
		Group A	−1.79 *	−2.87	−0.70	0.55	−0.38 *				
		Group B	−1.71 *	−2.79	−0.63	0.54	−0.37 *				
		Age	−0.02	−0.10	0.07	0.04	−0.04				
**Physical fitness**	**AC (Rep)**	Δ2 Model						0.11	0.08	3.70	0.015
		Constant	−4.94	−13.42	3.55	4.27					
		Group A	2.39 *	0.84	3.93	0.78	0.36 *				
		Group B	1.52	−0.02	3.06	0.77	0.24				
		Age	0.06	−0.06	0.18	0.06	0.10				
	**BS (cm)**	Δ2 Model						0.13	0.10	4.48	0.006
		Constant	−1.89	−8.43	4.64	3.29					
		Group A	2.11 *	0.92	2.30	0.60	0.41 *				
		Group B	1.47 *	0.29	3.65	0.60	0.29 *				
		Age	0.02	−0.08	0.11	0.05	0.04				
	**SR (Rep)**	Δ2 Model						0.11	0.08	3.48	0.019
		Constant	−4.24	−12.80	4.31	4.30					
		Group A	2.15 *	0.60	3.71	0.78	0.33 *				
		Group B	1.83 *	0.28	3.38	0.78	0.28 *				
		Age	0.06	−0.06	0.19	0.06	0.10				
	**CS (cm)**	Δ2 Model						0.24	0.21	8.96	<0.001
		Constant	1.13	−9.55	11.81	5.37					
		Group A	4.18 *	2.23	6.12	0.98	0.47 *				
		Group B	4.59 *	2.65	6.52	0.97	0.52 *				
		Age	−0.03	−0.18	0.13	0.078	−0.03				
	2ST (Rep)	Δ1 Model						0.11	0.08	3.68	0.015
		Constant	−14.15	−52.57	24.27	19.33					
		Group A	6.86	−0.14	13.85	3.52	0.23				
		Group B	10.85 *	3.88	17.81	3.50	0.37 *				
		Age	0.19	−0.37	0.74	0.28	0.07				
		Δ2 Model						0.27	0.25	10.87	<0.001
		Constant	−11.00	−44.99	22.99	17.10					
		Group A	14.75 *	8.56	20.94	3.11	0.51 *				
		Group B	15.58 *	9.43	21.74	3.10	0.54 *				
		Age	0.08	−0.41	0.57	0.25	0.03				

* represents *p* < 0,05 for delta based on ANOVA for repeated measures; BMI—Body Mass Index; BM—Basal metabolism; W—Water; VF—Visceral fat; BOM—Bone mass; LM—Lean mass; FM—Fat mass; AC—Arm curl; BS—Back scratch; SR—Sit and reach; CS—Chair stand; 2 ST—2 min step test; M1—first moment of evaluation; basal level, M2—second moment of evaluation; M3—third moment of evaluation, at the end of study. Note. Model = “Enter” method in SPSS Statistics; B = unstandardized regression coefficient; CI = confidence interval; LL = lower limit; UL = upper limit; SE B = standard error of the coefficient; β = standardized coefficient; R^2^ = coefficient of determination; ΔR^2^ = adjusted R^2^.

## Data Availability

Data are available under request to the contact author.
